# Maladie de Horton révélée par une dyspnée

**DOI:** 10.11604/pamj.2015.20.248.4656

**Published:** 2015-03-16

**Authors:** Madiha Mahfoudhi, Habiba Mamlouk, Sami Turki, Adel Kheder

**Affiliations:** 1 Service de Médecine Interne A, Hôpital Charles Nicolle, Tunis, Tunisie

**Keywords:** Maladie de Horton, dyspnée, alvéolite lymphocytaire, corticoïdes, Horton disease, dyspnea, lymphocytic alveolitis, corticosteroids

## Abstract

Les manifestations pleuro-pulmonaires de la maladie de Horton sont rares et peu connues. Elles peuvent être inaugurales, à l'origine d'un retard à la prise en charge si elles sont méconnues. Il s'agissait d'un patient âgé de 75 ans, admis pour une dyspnée, une toux chronique et une fièvre. Il a reçu une antibiothérapie et a bénéficié d'une fibroscopie bronchique avec lavage broncho-alvéolaire à la recherche d'un germe, qui a révélé plutôt, une alvéolite lymphocytaire. L’évolution était marquée par la persistance des signes cliniques et du syndrome inflammatoire biologique. Un angio-scanner thoracique et une échographie cardiaque étaient sans anomalies. Une origine cardiaque, musculaire, hématologique, néoplasique, vasculaire ou métabolique de la dyspnée a été éliminée. Une maladie de Horton a été évoquée. La biopsie de l'artère temporale a confirmé le diagnostic d'une maladie de Horton. L’évolution sous corticothérapie était marquée par la disparition des signes cliniques et biologiques. Les manifestations pleuro-pulmonaires au cours de la maladie de Horton sont rares, et classiquement, rarement révélatrices de la maladie. La dyspnée peut initialement égarer le diagnostic vers d'autres étiologies notamment infectieuses. Le but de ce travail est d'insister sur le fait que la connaissance de ces différentes manifestations respiratoires au cours de la maladie de Horton (toux persistante, dyspnée, épanchement pleural) est utile au clinicien afin de prescrire une corticothérapie, chez un sujet le plus souvent âgé ayant un état fébrile et inflammatoire prolongé, permettant ainsi d’éviter l'apparition de complications.

## Introduction

La maladie de Horton est une artérite inflammatoire giganto-cellulaire, touchant électivement les artères de gros calibre, et en particulier les branches de l'artère carotide externe. L'atteinte artérielle peut toucher d'autres troncs et s'exprimer par une symptomatologie variable en fonction des territoires concernés. L'atteinte respiratoire est rare au cours de la maladie de Horton. Elle peut se manifester par une toux chronique, une dyspnée, un épanchement pleural ou une pneumopathie interstitielle. Nous rapportons l'observation d'un patient âgé de 75 ans chez qui une dyspnée a permis de révéler une maladie de Horton. Le but de ce travail est d'insister sur le fait que la maladie de Horton dans sa forme respiratoire pose un problème de diagnostic différentiel avec les infections pulmonaires, les néoplasies et les autres vascularites touchant le poumon d'où un retard diagnostique et thérapeutique avec un risque de complications vasculaires graves.

## Patient et observation

Il s'agissait d'un patient âgé de 75 ans, non tabagique, admis en raison d'une fièvre et d'une dyspnée de stade II de NYHA et d'une toux sèche évoluant depuis 3 mois. L'examen clinique n'a objectivé qu'une fièvre à 38,7°C avec une auscultation pulmonaire normale. L'examen neuro-musculaire était sans anomalies. Les pouls temporaux étaient discrètement diminués. Les gaz du sang ont révélé une discrète hypoxie avec une normocapnie et un PH normal. L’électrocardiogramme n'a pas noté d'anomalies. L'examen biologique a révélé un syndrome inflammatoire biologique avec une anémie normochrome normocytaire (hémoglobine à 11,5 g/dl). La radiographie de thorax était normale. Une origine infectieuse a été évoquée devant le terrain, la dyspnée et la fièvre, d'où la prescription d'une antibiothérapie à large spectre. L’évolution était marquée par la persistance des signes cliniques et biologiques. Le bilan infectieux était négatif. L'examen stomatologique et ORL étaient sans anomalies. Les hémocultures, l'ECBU et la recherche de BK dans les crachats étaient négatifs. Le Quantiferon test était négatif. Une échographie abdominale était normale. Un angio-scanner thoracique et une échographie cardiaque trans-thoracique et trans- œsophagienne étaient sans anomalies. Le scanner abdomino-pelvien était normal. Le bilan immunologique était négatif. Le dosage des marqueurs tumoraux était normal. Les explorations fonctionnelles respiratoires (EFR) ont révélé un discret syndrome restrictif avec une diminution minime de la capacité pulmonaire totale et de la capacité vitale. La DLCO était aussi discrètement diminuée. La fibroscopie bronchique avec un lavage broncho-alvéolaire n'a pas révélé de germes, mais plutôt, une alvéolite lymphocytaire isolée avec un rapport CD4 + /CD8+ supérieur à 5. La recherche de cellules néoplasiques dans le liquide bronchique était négative. La biopsie bronchique n'a pas révélé de signes de malignité, il y'avait des signes d'inflammation non spécifiques.

Le diagnostic d'une maladie de Horton a été évoqué. La biopsie de l'artère temporale a confirmé le diagnostic de cette maladie en retrouvant un infiltrat lymphocytaire prédominant dans le média avec des granulomes à cellules géantes, associés à une destruction de la limitante élastique interne. Plusieurs signes évoquant la maladie de Horton (céphalées, claudication de la mâchoire, hypersensibilité du cuir chevelu, troubles visuels, arthralgies rhizoméliques) manquaient à l'interrogatoire. L'examen ophtalmologique a montré uniquement une presbytie. La corticothérapie, débutée à la dose de 1 mg/kg/jour, a permis une disparition complète de la toux et de la dyspnée. Le périmètre de marche s'est nettement amélioré en quelques jours. Le syndrome inflammatoire biologique a également régressé. Les GDS se sont normalisés ainsi que les EFR et la DLCO. Le patient n'a pas présenté de récidive de sa symptomatologie initiale avec un recul évolutif de 7 mois.

## Discussion

Le poumon peut être touché dans plusieurs maladies systémiques et vascularites. Cependant, l'atteinte pulmonaire est rare au cours de la maladie de Horton. Le diagnostic reste difficile devant des formes atypiques à début inhabituel tel qu'un tableau respiratoire sans autres signes typiques de la maladie [1].

Notre observation illustre une difficulté diagnostique de la maladie de Horton puisque l'atteinte respiratoire (toux, dyspnée et alvéolite lymphocytaire) était au premier plan sans signes anamnestiques orientant vers cette maladie. Une recherche étiologique de la dyspnée a été réalisée chez notre patient. Il n'avait pas d'embolie pulmonaire. Une origine neuro-musculaire a été éliminée. L'anémie était modérée ne pouvant pas expliquer cette dyspnée. Une origine métabolique telle qu'une acidose métabolique a été aussi éliminée chez lui. Par ailleurs, la fonction cardiaque était conservée. Le contexte fébrile et inflammatoire orientait vers une origine respiratoire infectieuse, néoplasique, auto-immune ou une vascularite, mais tout le bilan étiologique était négatif. Vu l'association de la fièvre, du syndrome inflammatoire biologique et de la discrète diminution des pouls temporaux, le diagnostic d'une maladie de Horton a été évoqué et c'est la biopsie de l'artère temporale qui a permis de confirmer ce diagnostic.

Les manifestations respiratoires spécifiques de la maladie de Horton sont rares (environ 10% des cas), rarement révélatrices (4% des cas), caractérisées par des symptômes banals (toux, dysphonie, dyspnée paroxystique), et radiologiquement par des épanchements pleuraux, des opacités parenchymateuses ou exceptionnellement des embolies pulmonaires [1, 2]. La toux est la manifestation inaugurale la plus fréquente, pouvant égarer le diagnostic vers une affection purement broncho-pulmonaire ou ORL, infectieuse, néoplasique ou systémique. Elle était présente chez 21 patients (8%) parmi une série de 260 malades [2]. Il s'agissait d'une toux sèche, quinteuse, irritative, diurne et nocturne, insomniante, parfois résistante aux antitussifs mais cédant très rapidement au traitement corticoïde. Aucun signe évident de maladie de Horton n'a été trouvé initialement, en dehors d'une fièvre modérée et d'un syndrome inflammatoire. Le retard diagnostique a été de trois mois en moyenne [2]. Le diagnostic a été porté, suite à la survenue secondaire de céphalées (11 cas), d'un tableau de pseudopolyarthrite rhizomélique (cinq cas), de troubles visuels (un cas) ou de l'association de ces signes. La biopsie de l'artère temporale, réalisée dans tous les cas, était positive 12 fois [2]. La toux peut s'associer aux manifestations ORL et stomatologiques de la maladie de Horton (claudication de la mâchoire, otalgie, oedème de la face, dysphagie, douleur pharyngienne, trismus, dysphonie, glossodynie) [1, 3]. L’étude de Larson et al. [4] a montré dans une série de patients atteints d'une maladie de Horton une fréquence de 8%, avec une toux véritablement précessive des autres signes de la maladie de Horton dans 4% des cas. Le mécanisme de cette toux n'est pas connu: il pourrait être correspondre à la vascularite des artères pharyngées, laryngées ou pulmonaires péri-bronchiolaires [5]. L’évolution de la toux est parallèle à celle de la maladie, rapidement régressive sous corticothérapie et la rechute est possible lors de la décroissance ou l'arrêt du traitement [2]. La présence d'une dyspnée comme c'est le cas de notre patient, est en revanche, plus rarement décrite, égarant le diagnostic vers une origine cardiaque, une embolie pulmonaire, une origine infectieuse, une cause néoplasique ou une autre vascularite, responsable d'un retard diagnostique et thérapeutique.

Il a été rapporté un cas de dyspnée paroxystique à prédominance nocturne associée à une toux avec des signes cliniques congestifs droits sans argument pour une origine cardiaque. Deux observations ont présenté des accès dyspnéiques comparables [4, 6]. Les EFR montraient un syndrome obstructif ou une hyper-réactivité bronchique non spécifique. La régression des signes cliniques et la normalisation des EFR sous corticothérapie allaient dans le sens d'une atteinte spécifique de la maladie qui pourrait être liée à un bronchospasme en rapport avec l'atteinte artérielle inflammatoire [7]. Une observation d'une patiente âgée de 73 ans a été rapportée [8] ayant une toux sèche et une dyspnée de stade III de NYHA évoluant depuis 6 mois avec un syndrome inflammatoire biologique. Aucun signe d'artérite temporale n’était présent à l'interrogatoire. Le bilan cardiologique, infectieux et carcinologique était négatif. Les explorations fonctionnelles respiratoires ont mis en évidence un syndrome obstructif modéré. Le scanner thoracique retrouvait des nodules pulmonaires supra-centimétriques alors qu'il était normal chez notre patient. Ces nodules avaient un caractère hypermétabolique au PET scan [8]. Le PETscan n'a pas été réalisé dans notre cas. Le lavage broncho-alvéolaire retrouvait une alvéolite lymphocytaire comparable à ce qui a été retrouvé chez notre malade. La biopsie de l'artère temporale a confirmé le diagnostic de maladie de Horton [8]. La corticothérapie, débutée à la dose de 0,75 mg/kg par jour, a permis une disparition complète de la toux et de la dyspnée [8]. La tomodensitométrie thoracique a montré la disparition des nodules au terme de 2 mois de traitement [8].

L'atteinte parenchymateuse est très rare et décrite de façon inaugurale ou à l'occasion d'une rechute de la maladie après réduction ou arrêt de la corticothérapie [9]. Notre patient n'avait pas ni des signes cliniques, ni radiologiques en faveur d'une atteinte parenchymateuse. L'atteinte interstitielle est prédominante de type réticulée ou réticulo-micronodulaire à prédominance basale [9, 10]. L'atteinte macro-nodulaire peut prendre un aspect pseudotumoral [11]. Un syndrome inflammatoire est présent dans tous les cas avec une vitesse de sédimentation moyenne à la première heure de 98 mm (60–130) et une CRP moyenne de 131 mg/L (60–210) dans une série de 8 patients atteints d'une maladie de Horton avec des manifestations respiratoires inaugurales [1]. Différentes lésions radiologiques étaient constatées dans 5 dossiers dans cette série [1] à type d'un épanchement pleural ou un épaississement pleural, un aspect réticulo-micronodulaire et des plages de verre dépoli, une condensation parenchymateuse, un épaississement pleural bilatéral et des opacités parenchymateuses macro-nodulaires bilatérales évocatrices d'un lâcher de ballons.

Le PET scan peut mettre en évidence une fixation du 18-FDG au niveau de la paroi aortique et des troncs supra-aortiques [8], il n'y a pas de données spécifiques concernant l'atteinte pulmonaire de la maladie de Horton [1]. L’échographie des artères temporales, peut avoir un intérêt diagnostique en montrant une inflammation de l'aorte ou de ses gros troncs [1]. Le profil des EFR est rarement rapporté dans la littérature: syndrome obstructif, syndrome restrictif [10] ou diminution du transfert du CO [6, 12]. Le traitement corticoïde normalise l'examen [13]. La fibroscopie bronchique et le LBA ont un rôle essentiel pour l'enquête bactériologique et la recherche d'une néoplasie bronchique même en absence de signes radiologiques pathologiques. L'aspect macroscopique montre une bronchite inflammatoire. La cytologie du LBA est polymorphe avec une prédominance macrophagique dans quelques observations [7, 12, 14] ou une alvéolite lymphocytaire T4 [15], qui a été retrouvée dans notre cas. L'apport diagnostique des biopsies réalisées lors de l'examen fibroscopique bronchique est faible et retrouve des signes inflammatoires non spécifiques mais permet d’éliminer une néoplasie ou une autre vascularite (maladie de Wegener). Des biopsies bronchiques distales ont montré un granulome inflammatoire polymorphe en rapport avec une maladie de Horton dans deux observations [12, 16]. Une observation rapportait une biopsie pulmonaire chirurgicale de lésions nodulaires excavées montrant des granulomes avec des cellules géantes et des lésions de nécrose entourant des vaisseaux de toutes tailles [11]. La bonne évolution clinique et biologique sous corticothérapie et l'absence de récidive avec un recul de 4 ans sont en faveur de ce diagnostic. La prednisone, prescrite entre 0,5 et 1 mg/kg/j, permettait la disparition des signes cliniques respiratoires au cours de la maladie de Horton dans tous les cas [1, 4, 5, 6, 7].

L’évolution était comparable dans notre cas. La normalisation des images pulmonaires radiographiques était constatée entre 3 semaines et 3 mois [1]. Dans plusieurs cas publiés, il n'a pas été observé de récidive de la symptomatologie respiratoire au cours de la décroissance de la corticothérapie [4–7]. Une cortico-dépendance à dose inférieure à 10 mg/j de prednisone était notée dans certains cas, n'ayant cependant pas nécessité l'adjonction d'un traitement immunosuppresseur pour contrôler les symptômes [1]. La réponse à la corticothérapie est essentielle. La cortico-résistance précoce ainsi que la nécessité d'adjoindre un traitement immunosuppresseur doit mettre en doute le diagnostic d'une maladie de Horton [1].

## Conclusion

Bien que rarement documentée, l'atteinte respiratoire de la maladie de Horton peut être une dyspnée en rapport avec une alvéolite lymphocytaire riche en CD4+. La maladie de Horton doit alors être recherchée par un interrogatoire et un examen physique dirigés surtout chez un patient âgé fébrile ayant une dyspnée inexpliquée. Une biopsie de l'artère temporale doit être aussi proposée pour éviter un retard diagnostique parfois responsable de complications vasculaires graves et irréversibles.
